# Myofilament dysfunction in diastolic heart failure

**DOI:** 10.1007/s10741-023-10352-z

**Published:** 2023-10-14

**Authors:** Anahita Aboonabi, Mark D. McCauley

**Affiliations:** 1 Division of Cardiology, Department of Medicine, College of Medicine, University of Illinois at Chicago, 840 S. Wood St., 920S (MC 715), Chicago, IL 60612 USA; 2 Jesse Brown VA Medical Center, Chicago, IL USA; 3 Department of Physiology and Biophysics and the Center for Cardiovascular Research, College of Medicine, University of Illinois at Chicago, Chicago, IL USA

**Keywords:** Cardiomyocyte, Diastolic dysfunction, Myofilament, Relaxation

## Abstract

Diastolic heart failure (DHF), in which impaired ventricular filling leads to typical heart failure symptoms, represents over 50% of all heart failure cases and is linked with risk factors, including metabolic syndrome, hypertension, diabetes, and aging. A substantial proportion of patients with this disorder maintain normal left ventricular systolic function, as assessed by ejection fraction. Despite the high prevalence of DHF, no effective therapeutic agents are available to treat this condition, partially because the molecular mechanisms of diastolic dysfunction remain poorly understood. As such, by focusing on the underlying molecular and cellular processes contributing to DHF can yield new insights that can represent an exciting new avenue and propose a novel therapeutic approach for DHF treatment. This review discusses new developments from basic and clinical/translational research to highlight current knowledge gaps, help define molecular determinants of diastolic dysfunction, and clarify new targets for treatment.

## Introduction

Heart failure (HF), a primary cause of mortality in the USA, is a chronic progressive disorder in which the heart is incapable of pumping (systolic) or filling (diastolic) sufficiently. HF occurs due to either systolic or diastolic dysfunction, and many patients have a considerable overlap of both systolic and diastolic dysfunction. Greater than 50% of heart failure patients have impaired relaxation or predominant abnormalities in diastolic function, with relatively normal or minor depression in systolic ventricular performance; this condition is identified as diastolic heart failure (DHF) [1, 2].

DHF is a clinically distinct syndrome from systolic heart failure (SHF) with specific morphologic and functional changes [3]. Patients with SHF exhibit a distinctive metabolic profile, which becomes further pronounced when coupled with comorbidities like diabetes and kidney dysfunction. DHF is linked to elevated inflammation and oxidative stress markers, compromised lipid metabolism, heightened collagen production, and a reduction in nitric oxide signaling [4]. DHF occurs when the ventricle is unable to accommodate sufficient blood volume to maintain appropriate stroke volume during diastole despite normal diastolic pressure [5]. These abnormalities develop because of elevated ventricular stiffness and/or impaired ventricular relaxation, distinguished by decreased contraction velocity, lengthy relaxation, elevated filling pressure, and reduced cardiac output [6].

Although considerable progress has been made in managing and controlling systolic heart failure, the mechanisms contributing to DHF remain insufficiently investigated. Thus, DHF-specific treatments have lagged because of a gap in knowledge of the molecular and biochemical mechanisms leading to myocardial functional and structural modifications in this disorder. To address this knowledge gap, the current review discusses the pathophysiologic mechanisms causing diastolic dysfunction (DD) and the molecular mechanisms that control relaxation at the cardiomyocyte level. We specifically focus on mechanisms regulating cardiac relaxation which can promote DHF.

## Pathophysiology of diastolic dysfunction

The cardiac cycle involves two phases: (1) diastole, in which the heart relaxes and refills with blood; and (2) systole, characterized by robust cardiac contraction [7]. One cardiac cycle at a regular heart rate of 75 beats per minute and under resting conditions lasts 0.8 s; systole occupies one third and diastole two thirds of the cardiac cycle duration. However, during intensive muscle work, the duration of diastole decreases much more and usually lasts for approximately half a cardiac cycle time at the regular resting heart rate [8]. In DHF, there is a change in the balance of left ventricular filling pressures, causing them to increase unevenly in proportion to the magnitude of LV dilation. This change can result in an inappropriately rapid heart rate (tachycardia), reduced ability of the ventricle to relax and fill during the diastolic phase of the cardiac cycle (ventricular diastolic compliance), and/or compromised relaxation of the ventricle itself [9].

Both structural and biochemical modifications in cardiomyocytes are responsible for impaired ventricular relaxation. In addition, compliance and impaired relaxation can be caused by extra-cardiomyocyte factors in the extracellular matrix surrounding the cardiomyocytes and hormones regulating both structure and function [9]. Most often, DD is linked to cardiac hypertrophy and stiffening of ventricular tissue, which can cause the inability of the heart muscle fibers to return to regular size and inadequate ventricular filling volume under low pressure [10].

Changes in any of the intrinsic characteristics of cardiomyocytes, such as calcium homeostasis, myofilament function, cardiac metabolism, and cytoskeletal structure, can cause irregularities in both active relaxation and passive stiffness. Although impaired filling can be caused by anatomical abnormalities that delay filling, the current review will focus on decreased ventricular compliance resulting from cytoskeletal dysfunction. The comprehensive studies have adequately addressed other crucial elements of DHF, including cardiac metabolism [11], the renin–angiotensin axis [12], mitochondrial function [13], and animal research [14]. Within the subsequent sections, we will delve into several pivotal processes associated with cardiac relaxation.

## Reduced intracellular calcium

**Fig. 1:**
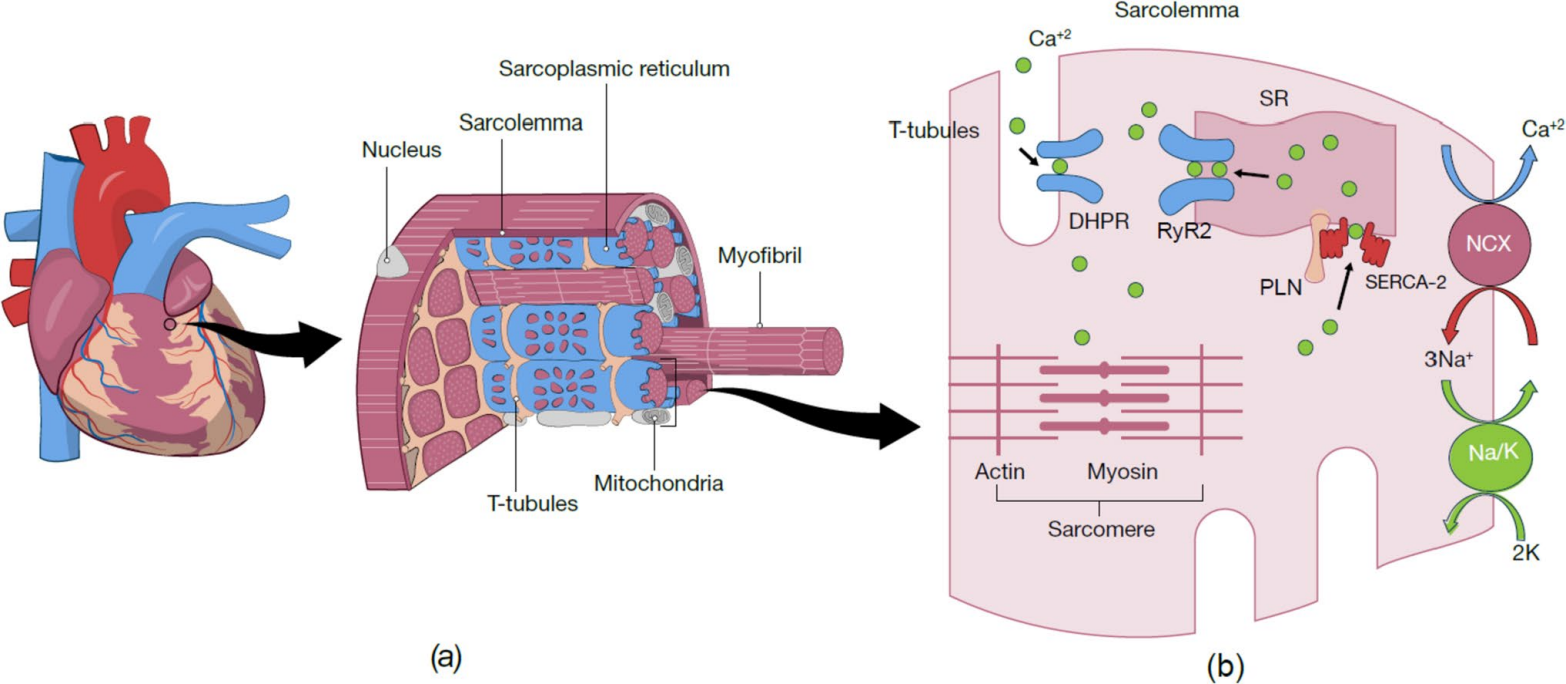
**a** Hierarchical scheme of cardiac structures. Bundles of myofibrils group together to form heart tissue. A bundle of myofibrils wrapped by sarcolemma and composed of the repeats of sarcomeres. SR establishes a mesh-like structure to cover the myofibers. Invagination of the sarcolemma, known as the T-tubule, is located transversely to the sarcomere and forms close contact with the SR. Mitochondria located next to the myofibers along the t-tubules and SR. **b** Intracellular Ca^2+^ cycling in cardiomyocytes. After depolarizing the sarcolemma, a small quantity of Ca^2+^ enters the sarcoplasm via the DHPR of the T-tubule, activating a rapid and large influx of Ca^2+^ release from inside SR via the RyR2. NCX is the main mechanism by which Ca^2+^ is released from cells. Activation of the SERCA2 pump regulated by its binding partner PLN promotes the reuptake of cytosolic Ca^2+^ into the SR. Ca^+2^, calcium; DHPR, dihydropyridine receptor; K, potassium; Na/K, Na+/K+-pump membrane receptor; NCX, sodium–calcium exchanger; PLN, phospholamban; SERCA-2, sarco/endoplasmic reticulum Ca^2+^-ATPase; SR, sarcoplasmic reticulum

Any process that interferes with calcium removal from the cytosol or cross-bridge detachment can potentially delay cardiac relaxation [7]. Depolarization of the plasma membrane is the initial phase of the cardiac cycle that opens L-type voltage-gated, dihydropyridine-sensitive sarcolemmal calcium channels known as dihydropyridine receptors to the influx of calcium into the myocyte [15]. The process involves the release of calcium from the sarcoplasmic reticulum (SR) through a channel called the cardiac ryanodine receptor (RyR2), facilitated in part by the close proximity between sarcolemmal calcium channels and RyR2 (as shown in Fig. 1) [15]. The sarcoplasmic reticulum handles calcium transportation within the cell and maintains the calcium concentration during the contraction–relaxation cycle. This process is crucial in regulating the contraction and relaxation of cardiac muscle [16–18]. Also, this relationship is supported by a mouse model study, which demonstrated that amplifying the presence of the α1-subunit of the L-type calcium channel (α1CTG) leads to dilated cardiomyopathy. This condition exhibits systolic and diastolic heart failure symptoms by the time the mice reach 4 months of age [19].

Increased concentration of intracellular calcium serves as the internal signal initiating the activation of the contractile response within sarcomeres [20]. Consequently, the elevation in calcium levels promptly instigates the process of calcium removal, leading to the subsequent deactivation of the contractile machinery [21]. Calcium removal from the cardiomyocyte cytosol is essential to initiate relaxation [10]. This process is regulated by the action of a calcium pump (SERCA2) and the sarcolemmal sodium–calcium exchanger [9]. SERCA2 is a significant regulator of cardiac relaxation since it largely determines the removal rate of more than 70% of cytosolic Ca^2+^ in human cardiomyocytes [22].

Consequently, SERCA2 activity reduction is linked to impaired relaxation and LV hypertrophy in HF due to decreased gene expression and phosphorylation of its repressive modulatory protein phospholamban [23, 24]. In addition, a significant decline in SERCA2 expression in hearts causes immediate severe myocardial systolic and DD and death from HF. For example, *Serca2*^null^ mutant mice die in utero, which shows the importance of a significant reduction in SERCA2 for cardiac function in the adult mouse (Table 1) [25, 26].

**Table 1:** Animal models of myofilament mutations causing diastolic heart failure

**Protein**	**Gene**	**Protein Name**	**Sarcomere component**	**Animal model**	**Reference**
ATPase Sarco/endoplasmic reticulum Ca2+ transporting 2	ATP2A2	SERCA2	sarco(endo)plasmic reticulum	SERCA2 whole body (WB) knock out (KO) & cardiomyocyte-specific excision (CMs)	[25, 26]
Myosin heavy chain	MYH6	α-MyHC	Thick filament (Atria)	WB Heterozygous aMHC^403/+^ mice (strain 129/BS)	[37]
	MYH7	β-MyHC (myosin mutation R723G)	Thick filament (Cardiac ventricles; slow-twitch skeletal muscle)	mutant β-myosin heavy chain-glutamic acid^403^ transgenic rabbit model of human HCM	[40]
Myosin-binding protein C	MYBPC33	cMyBP-C	Thick filament	CMs MyBP-C(-/-, Ex3-10)	[84]
Myosin light essential chain	MYL3	MLC1SB	Thick filament	Cardio-specific Tg-Δ43 mice	[42]
Myosin light chain 2, regulatory, cardiac, slow	MYL2	MLC-2A	Thick filament	Transgenic Lys104Glu-RLC-cardiomycocyte-specific	[41]
Actin	ACTC	α-cardiac actin	Thin filament	Transgenic mice model mutation ACTC E99K	[43]
Troponin complex and tropomuosin	TNNT2	Troponin T	Thin filament	CMs Transgenic rats with mutated human cTnT	[54]
	TNNI3	Troponin I	Thin filament	CMs Tnl KO mice	[51, 52]
	TPM1	α-Tropomyosin	Thin filament	CMs FHC 180 α-TM trangenic mice46	[56]
Titin	TTN	Titin	Thick filament/Z-Disc	CMs Murince Mode (Ttn^ΔlAjxn^)	[87]
Myosin light chain kinase 3	MYLK3	cMLCK/MLCK	Phosphoryl of cardiac myosin heavy chains	WB knockout (Mylk3 ^wild/-^)	[65]
Myosin light chain phosphatase	PP1CB	PP1 catalytic subunit beta	Thin filament	Not available	-
	PPP1R12C	PP 1 regulatory subunit	Regulates the catalytic activity	Not available	-
Muscle LIM protein	CSRP3	cysteine and glycine rich protein 3	Z-Disc	*csrp3* knockout zebrafish	[91, 92]
Telethonin	TCAP	titin-cap	Z-Disc	Telethonin-deficient mice	[93]
Myozenin 2	MYOZ2	myozenin 2	Z-Disc	WB Mutant MYOZ2-P48 mouse	[95]
Vinculin	VCL	vinculin	Intercalated disc	CMs Vinculin-Δln20/21 Mouse:	[98]

As mentioned above, Ca^2+^ cycling alterations have been shown in the last two decades that are closely linked to heart failure. In that case, Ca^2+^ overload during diastole via RyR2 can compromise sarcoplasmic reticulum Ca^2+^ storage capacity, impairing systolic contractility and possibly diastolic cardiac function. There is much to discuss regarding the clinical consequences of “leaky RyR2” and possible therapeutic strategies to correct RyR2 dysfunction [27]; however, the current review focuses on the role of myofilaments.

## Role of myofilament proteins in diastolic dysfunction

Regulation of myofilament function plays a pivotal role in overseeing the extent and rate of cardiac relaxation. While the leading phase in relaxation begins with a reduction in intracellular Ca^2+^ concentration to initiate relaxation, ventricular relaxation is predominantly regulated by the biophysical properties of the myofilament proteins. These proteins include actin, myosin heavy chain, troponins, tropomyosin, and regulatory enzymes such as myosin light chain kinase (MLCK) and myosin light chain phosphatase (MLCP). A discussion of the contribution of each protein is included below.

### Actin and myosin

**Fig. 2:**
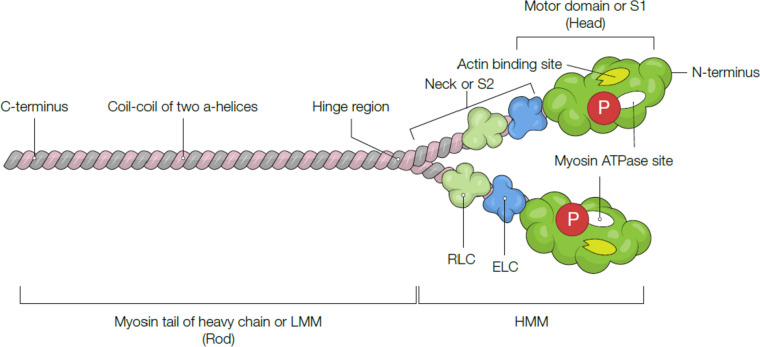
Schematic illustration of the cardiac β-myosin heavy chain (β-MHC). β-MHC contains two heavy chains (tail), an essential light chain (ELC), a regulatory light chain (RLC), a myosin neck chain (S2), and a head domain (S1). The two light chains of myosin are located near the myosin head and facilitate calcium-dependent force transduction by the myosin head domain. In addition, myosin ATPase can cause hydrolysis ATP to provide energy for actomyosin contraction

**Fig. 3:**
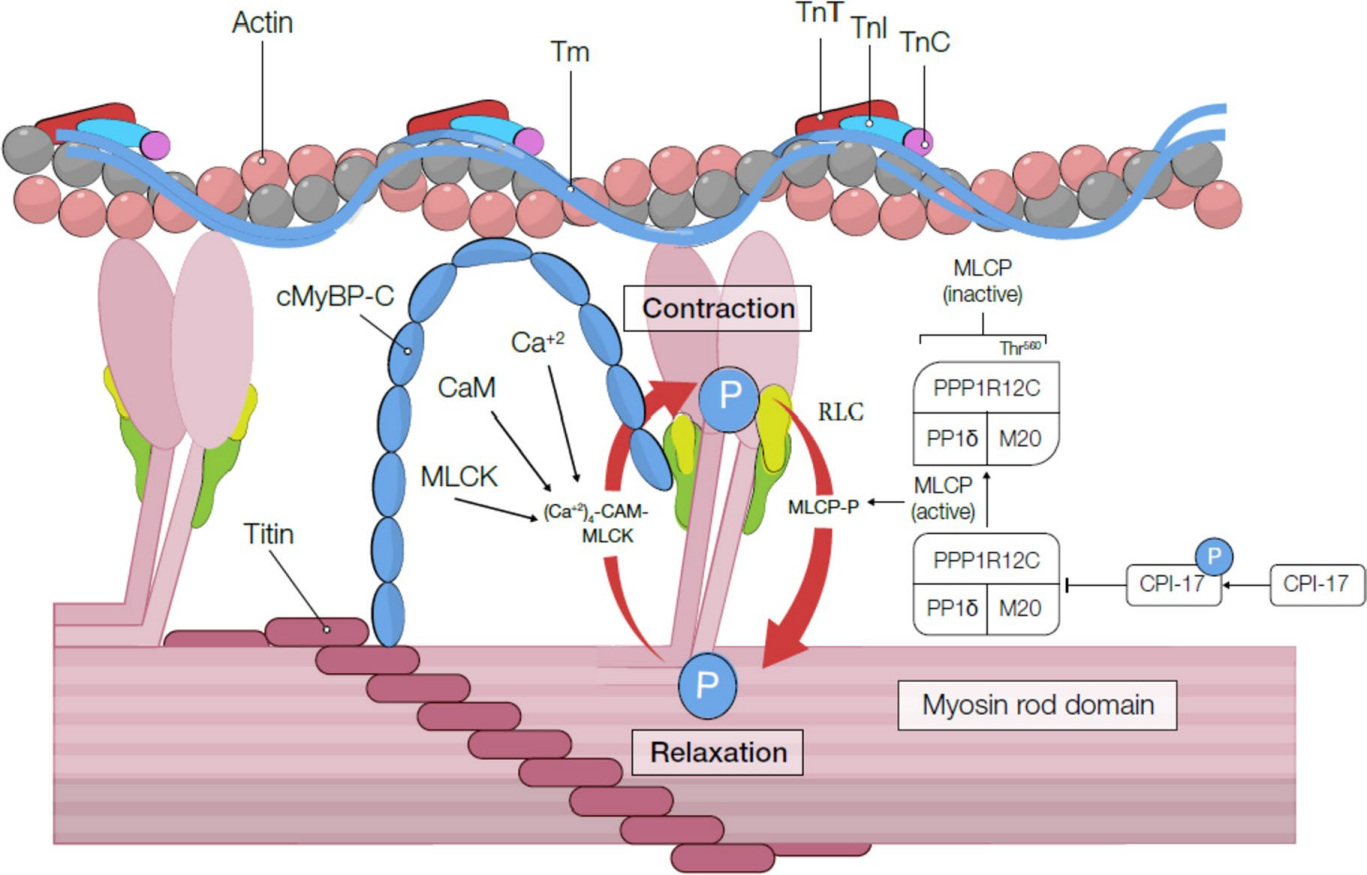
A schematic representation of contraction and relaxation with the interaction between troponin and other thin filament components. CaM, Calmodulin; cMyBP-C, cardiac myosin binding protein-C; CPI-17, C-kinase-potentiated protein phosphatase 1 inhibitor of 17 kDa; MLCK, myosin light-chain kinase; MLCP, myosin light-chain phosphatase; PKA, protein kinase A; TnI, troponin I; TnC, troponin C; TnT, troponin T; Tm, tropomyosin

To accomplish rapid and efficient contraction, cardiomyocytes are arrayed as a tubular assembly composed of chains of myofibrils (Fig. 1). The myofibrils consist of repeating segments of sarcomeres, an important contractile unit. Sarcomeres are made up of thick (myosin) and thin filaments (actin) [28, 29]. Myosin is composed of two globular myosin heads (S1), a myosin neck region (S2), and a tail or rod section [30] (Fig. 2). A tail domain consists of two heavy chains twisted together to shape a long coiled-coil α-helical tail domain. However, two round myosin heads develop from the N terminus, which is attached to the neck with two essential light chains (ELC) and the regulatory light chain (RLC) (Fig. 2) [28]. Thin filaments contain three main proteins: actin monomer, two strings of tropomyosin molecules, and troponin complex (Fig. 3).

Muscle contraction involves the interaction between the thick filament of myosin heads and the thin filament of actin subunits. However, relaxation occurs when molecular switches block this interaction on these filaments [31]. The sliding of myosin head on actin monomers produces the formation of “cross-bridges,” which causes heart contraction and generation of force [32, 33].

Actin–myosin interaction and force generation are fundamental to the pathophysiology of HF. Usually, the β-myosin isoform is expressed mainly in the ventricles, while the atria mostly express the α-isoform in the human heart [34]. Alterations in myofilament isoforms themselves can also delay relaxation. An illustration of this concept is comparing the α- and β-myosin heavy chain (MHC) isoforms, where the α-isoform is associated with a prolonged increase in force during contraction and relaxation phases [35]. However, this modification is observed mainly in larger mammals and fewer humans because the β-MHC isoform dominates under normal conditions.

Mutations in genes encoding myosin and actin are linked to various types of cardiomyopathies. Specific mutations in myosin can directly affect its interaction with actin. Among the most common causes of cardiomyopathy are mutations in myosin. A summary of known mutations in both actin and myosin is provided in Table 1 [36]. For instance, a mutation in the α-myosin heavy chain (α-MHC) at position 403 (arginine to glutamine) is associated with familial hypertrophic cardiomyopathy (FHC). In mice with the same missense mutation (αMHC^403^/^+^) as in humans, a model of this genetic disease shows diastolic dysfunction similar to human FHC [37].

Mutations in the β-cardiac myosin heavy chain (β-MyHC) gene (MYH7) can lead to ventricular wall hypertrophy and changes in diastolic filling volumes. For example, a mutation in the myosin heavy chain, R723G (MyHC723), reduces calcium sensitivity in cardiomyocytes. Calcium sensitivity, crucial for contractile function, is regulated independently from the actin regulatory protein complex [38, 39]. In addition, a transgenic rabbit model of mutant β-myosin heavy chain-glutamic acid at amino acid 403 also reduced myocardial contraction and relaxation [40].

Mutations in the Regulatory Light Chain (RLC) can impair ventricular relaxation, affecting indicators of left ventricular (LV) function like the E/A ratio (a measure of diastolic filling). This mutation can lead to energy inefficiency due to decreased contractile force and quicker ATP consumption [41]. The Myosin Essential Light Chain (ELC), encoded by the MYL3 gene, is essential for cardiac contraction. Transgenic mice expressing a mutation in this gene (Tg-D43) exhibit pathological cardiac hypertrophy [42].

Song et al. discovered that a mutation in the cardiac actin gene (Actin Alpha Cardiac Muscle 1, ACTC) in a transgenic mouse led to dilated cardiomyopathy. This condition was characterized by increased end-systolic capacity, end-diastolic pressure, and extended relaxation and contraction rates [43]. Over time, these symptoms could progress to apical hypertrophic cardiomyopathy, marked by heightened myofibrillar calcium sensitivity responsible for apical hypertrophy. This progression further leads to heart failure’s eventual development in mice and humans [43].

### Troponin complex and tropomyosin

The troponin complex regulates myofilament response to Ca^2+^ and cardiac muscle contraction. The hetero-trimeric troponin complex comprises three regulatory proteins: cardiac troponin T (cTnT), troponin I (cTnI), and troponin C (cTnC). Together with tropomyosin 1 (TPM1), a contractile protein, they are positioned on the actin filament [44]. cTnI, commonly used as a marker for myocardial damage, plays a crucial role in regulating cardiac function. cTnI is a protein that helps regulate the interaction between actin and myosin in the heart. It acts as an inhibitor to prevent the binding of myosin to actin, thereby stopping the formation of cross-bridges and allowing for the relaxation of the heart muscle [45].

In systole and at higher Ca^2+^ concentrations, cTnI goes through major changes both in conformation and position to be disconnected from actin–tropomyosin activation of the thin filament, allowing for a strong cross-bridge binding with myosin [46], linked to the force generation and elevated rate of ATP hydrolysis (Fig. 3). However, in diastole and lower Ca^2+^ concentrations, cross-bridge bindings are weakened or blocked from interacting with actin without generating force. cTnI plays a significant role in regulating the actin and tropomyosin complex on the actin filament. It attaches to tropomyosin and actin, forming a complex that helps regulate muscle contraction. The binding of cTnI to tropomyosin stabilizes the position of tropomyosin on the actin filament, preventing myosin from binding to actin and inhibiting muscle contraction. This interaction ensures proper muscle contraction and relaxation control, allowing the heart to function effectively [47, 48].

Troponin mutations have been shown to cause dilated cardiomyopathy and diastolic dysfunction in transgenic mice. The deficiency of cTnI, or modifications in cTnI, in pathological conditions, especially in the C terminus of cTnI, is connected to DD triggered by myofibril hypersensitivity to Ca^2+^ [49].

Mutations of cTnI in the region of PKA-targeted phosphorylation sites are associated with HF in which force-frequency modulation is lessened and afterload relaxation sensitivity rises in connection with reduced PKA TnI phosphorylation (Table 1) [50]. However, when cTnI is overexpressed or constitutively phosphorylated, it can impair heart relaxation [51]. Much of this mechanism is still unknown. Huang et al. conducted a study using a cardiac troponin I (cTnI) gene knockout mouse model created by targeting murine embryonic stem cells [52]. This model aimed to investigate the effects of troponin I deficiency on cardiac function and survival. The study results showed that mice lacking cardiac troponin I were born healthy with normal heart and body weight, as a fetal troponin I isoform compensated for the absence of cardiac troponin I. However, this compensation was temporary, and 15 days after birth, the expression of the compensatory isoform began to decline. This decline led to a troponin I deficiency, which resulted in a lethal form of acute heart failure. The mice died on day 18, demonstrating that the troponin I is necessary for normal cardiac function and survival [52]. A similar effect was observed in which mutations and cTnI deficiency caused DD and restrictive cardiomyopathies [53]. In addition, a human cTnT obliteration in transgenic rats exerts and mimics the phenotype of FHC with DD and arrhythmias [54]. These genetic modifications are distinguished by relatively mild and sometimes clinical hypertrophy but a high incidence of sudden death. Therefore, genetic testing may be necessary for this group [55].

In addition, mutations in ɑ-tropomyosin, an essential sarcomere component, regulate muscle contraction through calcium-mediated activation and can severely disrupt sarcomeric function (Table 1). Physiological analyses of mice with ɑ-tropomyosin mutation reveal functional differences in diastolic performance and increased calcium sensitivity, which can severely disrupt sarcomeric function. This mutation can cause ventricular hypertrophy, atrial enlargement, fibrosis, and culminating death in 5 months [56]. Despite the lethality of this mutation, there are no suggested treatments to modulate this activity.

### Cardiac myosin light-chain kinase

Myosin light-chain kinase (MYLK or MLCK) is a segment of a Ca^2+^/calmodulin (CaM)-dependent protein kinases group. It is a serine/threonine-specific protein kinase that regulates the light chain of myosin II phosphorylation [57]. Four distinct versions of the MYLK gene exist, each producing a specific MYLK isoform: MYLK1, MYLK2, MYLK3, and MYLK4 [58]. MLCK3 and MLCK4 are expressed in cardiac muscle based on their muscle type, known explicitly as cMLCK [59]. cMLCK is required for normal RLC phosphorylation and physiological cardiac operation [59]. cMLCK is essential in cardiomyocyte contraction [60] and is involved in efficiently coupling energy sources and force development [61]. Contraction is initiated with an influx of Ca^2+^ into the cardiac muscles from the sarcoplasmic reticulum (SR) and the extracellular space and binding to calmodulin [62]. Once Ca^2+^ levels are elevated, Ca^2+^ binds to calmodulin (CaM), which then Ca^2+^/CaM causes a conformation change in MLCK and activation (Fig. 3). Activated MLCK can increase the regulatory RLC phosphorylation of serine residue 19. Phosphorylated myosin and ADP and inorganic phosphate (Pi) can create connections called cross-bridges with actin. Releasing ADP and Pi triggers a power stroke that triggers muscle contraction [63]. This force leads the thin filament to glide past the thick filament and compresses the sarcomeres. Consequently, MLC phosphorylation lets myosin cross-bridges bind to the actin and enable contraction to initiate. Finally, the ATP binds to myosin and then hydrolyzes, which can release myosin from actin and repeat the process (Fig. 3).

Recent studies identified that cMLCK is associated with familial dilated cardiomyopathy. Heterozygous *Mylk3* knockout mice indicate a mild decrease in cardiac contractility by 4 months of age. These mice partially resemble humans with the heterozygous MYLK3 mutation, but the diminution in cardiac contractility was slighter [64]. Also, the outcomes on Tg-D166V mice recommended that a mutation-induced reserve of RLC phosphorylation might cause the development of cardiomyopathy phenotype [65].

Reduction of intracellular calcium concentration inactivates MLCK but does not stop muscle contraction since the MLC has been physically adjusted through phosphorylation and not through ATPase activity. Therefore, to halt muscle contraction, these changes need to be reversed. Dephosphorylation of the MLC and following termination of muscle contraction happens through the action of a second enzyme identified as myosin light-chain phosphatase (MLCP).

### Cardiac myosin light-chain phosphatase

**Fig. 4:**
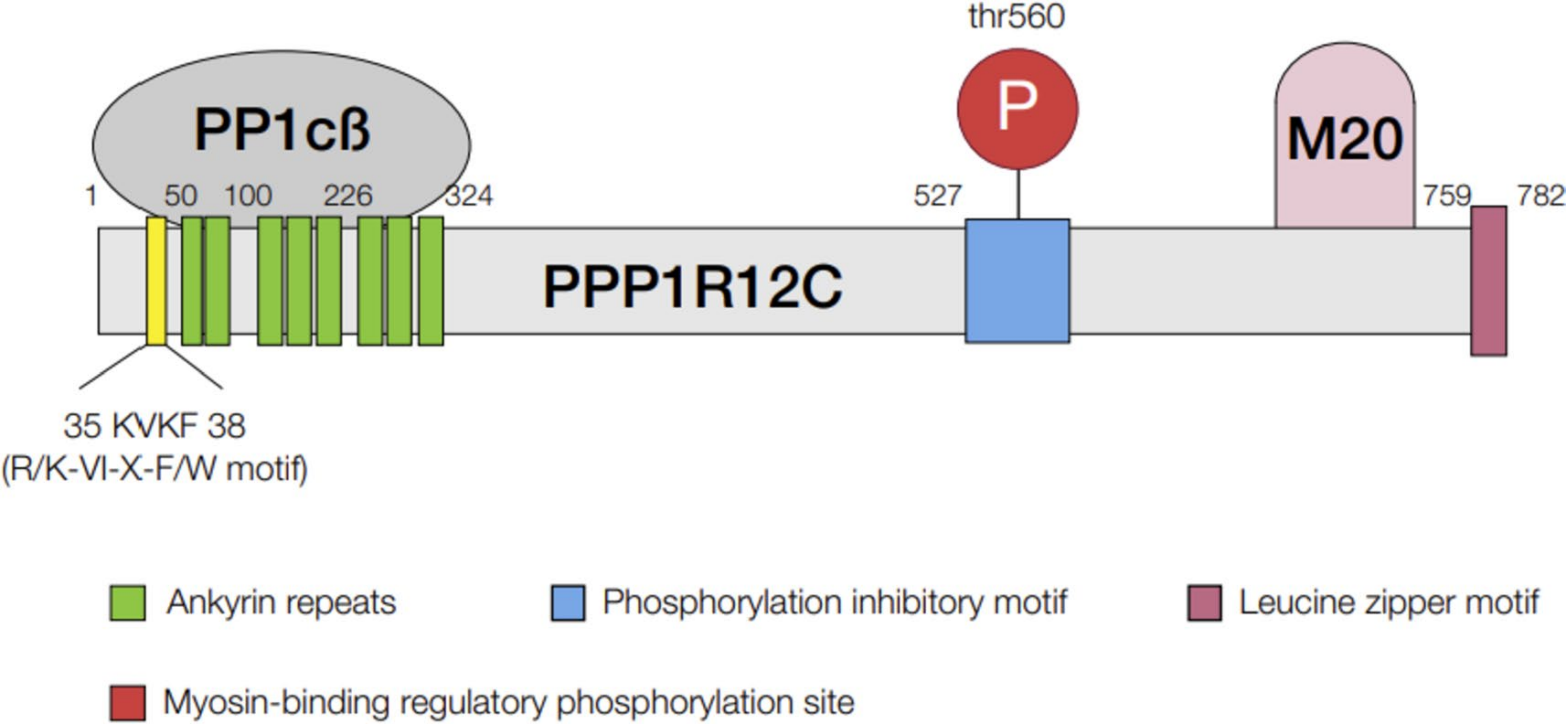
Subunit structure of cardiac heart myosin target phosphatase. KVKF motif, M20, 20 kDa small regulatory subunit; PP1cβ binding site; P, phosphorylation site; PPP1R12C, protein phosphatase 1 regulatory subunit 12C; PPPP1Cβ; protein phosphatase 1 catalytic subunit beta

Cardiac myosin light-chain phosphatase (cMLCP) is a serine (Ser)-threonine (Thr) phosphatase that is accountable for the dephosphorylation of the RLC and so regulates relaxation in myofilament [66]. The typical structure of MLCP is a holoenzyme with three following subunits: (1) a catalytic phosphoprotein phosphatase (PP1c) type-I Ser/Thr-phosphatase PP1c (37 kDa), (2) a sizeable regulatory subunit called myosin phosphatase target subunit (MYPT) or myosin-binding subunit (MBS), in the range of from 115 kDa in MYPT1 to 58 kDa to in MYPT3, and (3) a small accessory subunit of 20 kDa called M20 that is tightly bound MYPT with unknown function [67]. MYPT plays a strategic role in regulating the functional and physical integrity of the trimeric MLCP holoenzyme (Fig. 4).

The catalytic subunits of type 1 phosphatase (PP1C) are outcomes of three genes α (PPP1CA), β/δ (PPP1CB), and γ (PPP1CC). Of these isoforms, MYPT1 and MBS85 attach specifically to PP1cβ/ [68]. A sizeable regulatory subunit that was recently re-classified as an RRR1R1 contains the outcomes of five different genes, including MYPT1 (PPP1R12A), MYPT2 (PPP1R12B), MBS85 (PPP1R12C), MYPT3 (PPP1R16A), and TIMAP (PPP1R16B). These regulatory subunits share several preserved domains, including multiple ankyrin repeat domains and an RVxF motif for PP1c binding to mediate protein–protein interactions [69]. MYPT1, the regulatory subunit of MLCP, has a role in the phosphatase activity in smooth muscles. MYPT2 and MBS85 (PPP1R12C), other myosin-targeting subunits of MLCP, are the critical phosphatase in skeletal muscles and cardiac tissues [69, 70] and are likely to subcellular structures such as myofilaments. Despite the critical role of cMLCP in relaxation, few genetic models are available to study the effect of this enzyme’s consequence mutation and depletion in DHF. MLCP activity may alter the phosphorylation rate independent of MLCK activity. Thus, the activation of MLCP may upgrade relaxation. On the other hand, MLCP inhibition may also contribute to a worsening of cardiomyocyte contraction by increasing MLC phosphorylation to induce hypercontractility. Since the MLCP regulation in cardiomyocytes is mediated mainly by PP1C and PPP1R12C subunits without any significant overlap with other MYPT families [71], these two proteins may be involved in cardiomyocyte relaxation and, in general, contribute to DHF. In addition, the DHF model of a high-fat diet (HFD) with nitroarginine, as an inhibitor of nitric oxide synthase, is associated with endothelial dysfunction and reduction in MYPT1, thus altering MLC2v phosphorylation [72].

## Sarcomere lengthening

Another key modulator of cardiac relaxation is the direction and amplitude in which sarcomeric proteins move. Rapid post-systole relaxation is necessary to efficiently fill the left and right ventricles [66]. Conversely, the ventricular filling is impeded when relaxation occurs too slowly or incompletely, leading to clinical DHF [73].

Relaxation has been shown to accelerate significantly when a sarcomere length is lengthened at the end of the systole [70]. This is a Frank–Starling effect as it is a fundamental biophysical property of the myofilaments. Generally, an enlargement of the sarcomere may provide a slighter chance for the myosin head to bind to the actin-binding site compared to an isometric or shortening sarcomere based on an estimate of filament compliance on the requirement of fiber stiffness on sarcomere length [74, 75]. This phenomenon also explains why isovolumic relaxation is prolonged during early filling in patients with DD [76]. Once the myocardium relaxes and sarcomeres stretch, it may suggest a mechanical way to terminate other cross-bridge formations, regardless of a potentially activated thin filament [77]. Cardiac Myosin Binding Protein-C (cMyBP-C) (Fig. 3), a possible activator of the thin filament, is a regulatory protein positioned on a thick filament [78]. Mutations and modifications in the cardiac MyBP-C gene (MyBP-C^-^^/-^ knockout mice) are associated with DHF [79]. Since MyBP-C has been indicated to adjust the cross-bridge cycling kinetics and could change contraction–relaxation coupling by the amount and direction of expansion, the exact overall sarcomere size can influence myocardial relaxation [80]. Consequently, mutations in cMyBP-C have the potential to lead to DD [78, 81, 82]. Individuals carrying cMyBP-C mutations might display diastolic dysfunction, characterized by decreased relaxation velocity in the heart muscle. Importantly, this effect is observed regardless of hypertrophy [83]. A cardiac-specific mouse model that targets exons 3–10 (cMyBP-C(-/-, Ex3-10)) exhibits DD with a more significant *E*/*E*′ ratio similar to human patients [84]. In addition, cMyBP-C phosphorylation inhibits cardiac dysfunction linked to aging. This study presented that aging is associated with reducing cMyBP-C phosphorylation and deteriorating cardiac dysfunction, and cMyBP-C phosphorylation can adjust diastolic function [85].

Moreover, genetic obliteration of the I-band–A-band junction (IAjxn) in titin protein encoded by TTN in cardiac-specific *Ttn*^ΔIAjxn^ mice can cause HF with normal ejection fraction (HFNEF) showing higher filling pressures and lowered ventricular compliance [86]. Bull et al. showed that genetic removal of the I-band–A-band junction (IAjxn) in titin raises force on the spring region, leading to an HFNEF-like syndrome in the mouse [87]. In addition, the TtnΔIAjxn mouse model showed higher diastolic stiffness and lower exercise tolerance, similar to HFNEF symptoms observed in patients [88]. Titin is a massive protein in the thick filament that connects the Z and M lines in the sarcomere segment, causing force transmission to the Z line and friction in the I band section. Soetkamp et al. showed that increased titin phosphorylation at the junction of the Z-disk affects the myofilament structure and contractility performance and is associated with DD [89]. Also, higher PKCα activity is a crucial modulator of cardiomyocyte stiffening in diabetic hearts due to titin-based modifications [90].

In addition to titin, mutations in other sarcomere genes are also causative of human cardiomyopathies. Chang et al. showed that cysteine and glycine-rich protein 3 (CSRP3) play a role in cardiac stretch sensing. Genetic mutations in the CSRP3 gene can result in cardiomyopathies, paralleled to human patients with downregulation of CSRP3 showing HF [91, 92]. Telethonin whole-body knockout mice develop HF following biomechanical stress, owing at least in part to apoptosis of cardiomyocytes [93]. This effect may also affect human heart failure [94].

Ruggiero et al. demonstrated in a human study that calcineurin protein phosphatase 2B (PP2B) plays a pivotal role in the development of hypertrophic cardiomyopathy. Moreover, they highlighted the significance of mutations in myozenin 2 (MYOZ2) as calcineurin inhibitors, underscoring their contribution to the onset of cardiomyopathy. These findings shed crucial light on the disorder’s molecular mechanisms, enhancing our understanding of its pathogenesis and potentially guiding future therapeutic interventions [95].

Finally, Vinculin (Vcl), a membrane-associated protein expressed in intercalated disks, is a crucial structural part of forming costamere protein complexes. Vcl connects the actin to integrins on the cell surface of cardiomyocytes [96]. Vcl mutations have been linked with dilated and hypertrophic cardiomyopathies in human knockout, resulting in heart and brain defects during embryonic [97]. In addition, the knockout mice model of cardiomyocyte-specific Vcl (cVclKO) exhibited a significant reduction in membrane cortical stiffness because of the expanded lattice spacing, which might explain the systolic wall strains before the beginning of ventricular dysfunction [98].

In addition to the genetic model, other available surgical and metabolic mouse models of diastolic heart failure have already been addressed [99, 100]. Beyond the crucial role myofilaments play, other factors can also significantly contribute to the development of DHF, which will be discussed in the upcoming sections, shedding light on the intricate nature of DHF and its underlying mechanisms.

## Extracellular matrix

Abnormal extracellular matrix (ECM) remodeling contributes to diastolic dysfunction and impaired ventricular relaxation [7]. Changes in the architecture, composition, and distribution of the ECM play a central role in the pathophysiology of DHF [101, 102]. The ECM forms a dynamic network of molecules and proteins that offer structural support to cardiomyocytes [103]. ECM composition and organization alterations contribute to the impaired relaxation and increased stiffness characteristic of the disorder. Excessive deposition of collagen and other matrix proteins can lead to fibrosis, compromising the compliance of the myocardium during diastole [104, 105]. Changes in ECM properties can also influence the behavior of resident cardiac cells, including fibroblasts, cardiomyocytes, and endothelial cells, further impacting cardiac structure and function [106, 107]. The complex relationship between ECM and DHF has valuable therapeutic implications. By targeting ECM pathways, novel strategies can be developed to restore cardiac compliance, reduce fibrosis, and enhance prognosis for those with DHF.

Collagen, a fundamental structural protein in the ECM of the heart, plays a critical role in maintaining the integrity and function of cardiac tissue. Its dynamic balance, referred to as collagen homeostasis, is pivotal for maintaining appropriate cardiac structure and function [108]. Recent research has revealed a compelling connection between collagen homeostasis and the development of DHF [108]. In cases of DHF, the relaxation phase of the heart’s rhythm is compromised, leading to heightened stiffness in the cardiac muscle. This stiffness is frequently attributed to modifications in the composition and distribution of collagen throughout the myocardium. Disruptions in collagen synthesis, degradation, and cross-linking processes can induce fibrotic alterations that impede cardiac flexibility and relaxation [109, 110]. Understanding the intricate interplay between collagen homeostasis and DHF presents promising opportunities for therapeutic interventions aimed at reinstating normal cardiac function and enhancing patient outcomes.

## cGMP/PKG signaling

The cGMP/PKG signaling pathway has emerged as a significant player in DHF.

Numerous investigations have indicated that enhancing the pathways involving cyclic guanosine monophosphate (cGMP)-dependent protein kinase or protein kinase G (cGMP–PKG) holds significant potential as a target for enhancing diastolic function in patients with DHF [111]. The presence of inflammation disrupts the intricate local communication between endothelial cells and adjacent cardiomyocytes, significantly impacting the nitric oxide (NO)–cGMP–PKG pathway and NO production. This diminishing signaling pathway lays the groundwork for cardiomyocytes to undergo hypertrophy and elevate diastolic resting tension [112]. Delayed relaxation and increased diastolic stiffness as a predominant clinical feature of DHF have demonstrated positive responses to elevated PKG activity [113]. A recent study showed that the cardiac-specific deletion of STAT3 leads to the creation of a mouse model for DHF, mirroring clinical traits, partially achieved by impacting the cardiac levels of PKG [114]. Empagliflozin, by inhibiting the sodium-dependent glucose co-transporter 2, reduces inflammatory and oxidative stress, leading to an enhancement of the NO–sGC–cGMP pathway. This improvement results in heightened PKGIα activity, achieved by mitigating PKGIα oxidation and polymerization, ultimately leading to decreased pathological stiffness in cardiomyocytes [115].

On the other hand, nitrosative stress emerges as an overabundance of reactive nitrogen species (RNS), prominently including NO, a crucial signaling molecule renowned for its contributions to vasodilation and cardiac relaxation [116, 117]. Post-translational modifications of the sarcomeric protein, such as titin, are pivotal in enhancing cardiomyocyte stiffness, contributing significantly to diastolic dysfunction. Elevated nitrosative and oxidative stress, impaired NO availability, and suppressed cGMP and PKG signaling pathways initiate alterations in titin's structure and function. These changes, affecting titin isoform expression and phosphorylation patterns, lead to amplified cardiomyocyte stiffness [117]. Shifts in titin isoforms toward stiffer forms and disrupted phosphorylation patterns due to oxidative stress compromise titin’s compliance, collectively intensifying cardiomyocyte stiffness. This interplay between stress factors, signaling attenuation, and titin modifications highlights their complex role in shaping left ventricular diastolic stiffness, offering insights into diastolic dysfunction within the context of heart failure [103].

Nitrosative stress can also activate the unfolded protein response (UPR) within cells [118]. The UPR is a cellular signaling pathway activated in response to the accumulation of misfolded or unfolded proteins within the endoplasmic reticulum (ER), a cellular compartment responsible for protein folding and quality control [118]. Nitrosative stress can impact ER homeostasis and protein folding, leading to the induction of the UPR. Nitrosative stress can trigger the UPR through protein nitrosylation [119], ER disruption [120], calcium dysregulation [121], and induction of ER stress sensors [122]. Unraveling the intricate relationship between nitrosative stress, the unfolded protein response, and DHF holds promise for advancing the understanding of disease progression and identifying new therapeutic avenues to restore proper cardiac function and improve patient outcomes.

## Diagnostic

**Fig. 5:**
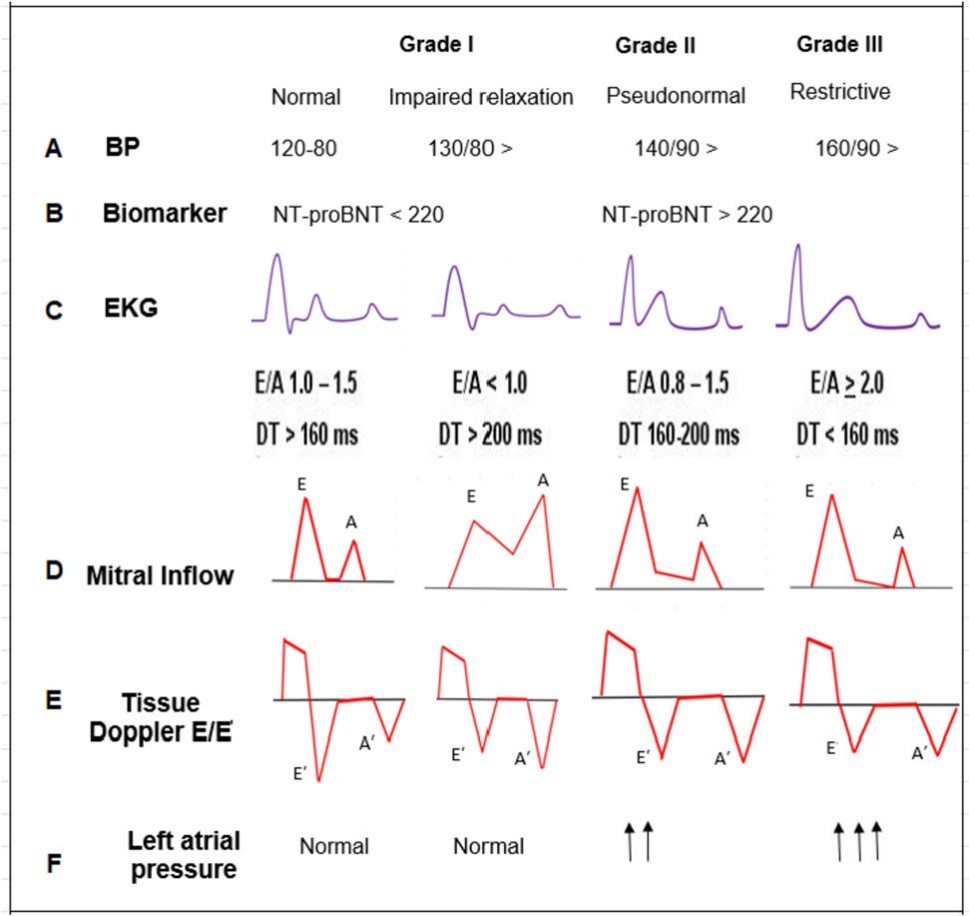
Characterization of diastolic dysfunction by echocardiography. BP, Blood pressure; EKG, electrocardiogram; TD, tissue Doppler; E, the velocity of blood flow across the mitral valve during early diastolic; A, velocity of blood flow across the mitral valve during atrial contraction; Aʹ, velocity of myocardial tissue relaxation during atrial contraction; Eʹ, the velocity of myocardial tissue relaxation during early diastole

As delineated above, DHF is the hemodynamic manifestation of impaired LV filling volume due to myocardium abnormalities identified in relaxation and stiffness of the impaired active ventricular relaxation due to cellular anomalies during diastole, producing a stiff ventricle, thereby increasing LV end-diastolic pressure. Based on a European Study Group guideline of diastolic HF, the concurrent presence of three following criteria can be considered for establishing a diagnosis of DHF: (1) indication of heart failure symptoms, (2) LV systolic function may be normal or slightly abnormal, and (3) LV relaxation function is abnormal due to diastolic stiffness, or filling diastolic capacity [123]. Based on this guideline, a patient who meets the following criteria is defined as a DHF (Fig. 5).

DHF is also related to increased age and other cardiovascular risk factors, such as diabetes and high blood pressure (BP). BP is the most common risk factor and the fundamental precursor of HF [124]. The risk for progressing HF in hypertensive in comparison to normal BP individuals is about twofold in men and threefold in women [125]. In patients with HF with normal ejection fraction, guidelines recommend hypertension with a target BP of 130/80 mmHg [126].

Diagnostic evidence of DHF can be acquired by invasive measurement of LV pressure during cardiac catheterization by measuring the timing and rate of the cardiac cycle [127, 128]. Cardiac catheterization, or catheter insertion into a heart chamber, is the latest method for indicating the characteristics of DHF [129]. However, because of the risks and costs of invasive hemodynamic assessment, it is not practical to diagnose DHF [130]. On the other hand, non-invasive imaging methods provided by echocardiography, with less risk than heart catheterization, are beneficial for showing diastolic abnormalities by evaluating myocardial tissue motion, LV dimensions, and filling dynamics [131–133]. The mitral inflow velocities are the most familiar by measuring the E and A waves corresponding to the blood flow velocity during LV relaxation and atrial contraction, respectively [134]. In normal diastolic function, E wave surpasses A wave velocity. However, with Grade I (impaired relaxation), A will exceed E and E/A ratio < 1.0, as the atrial contraction is more responsible for ventricular filling, and the deceleration time of E > 200 ms (Fig. 5). Therefore, if the *E*/*E*′ ratio indicates diastolic dysfunction, extra non-invasive examinations are essential to diagnose the evidence of diastolic LV dysfunction [135]. Also, electrocardiographic evidence of atrial fibrillation or plasma levels of atrial natriuretic peptide (ANP) and brain natriuretic peptide (BNP) could be other options [136, 137]. However, echocardiography has limitations associated with poor quality images, fewer details in pixels of images, geometric assumptions, and highly depends on the operator’s skills to accurately assess and calculate diastolic function [102].

Cardiac MRI evaluations are better than echocardiography in accuracy for evaluating DHF, especially in patients with cardiovascular symptoms, including hypertrophic cardiomyopathy, hypertension, and congestive heart failure [138]. In addition, MRI gives several parameters that evaluate heart function and morphology, hemodynamic parameters, myocardial contractility, and tissue characterization [139]. Therefore, the recent developments in MRI technology are supposed to develop in wider clinical adoption combined with other clinical practice techniques.

## Treatment

Although there have been notable improvements in managing DHF, the unfavorable outlook for patients underscores the need to investigate novel pathways for drug testing. These approaches should extend beyond conventional methods and instead concentrate on inventive techniques that address cardiomyocytes and myofilament function. Current treatment for DHF is based on reducing symptomatic using drugs such as β blockers, disopyramide, and non­dihydropyridine Ca^+2^ channel blockers. Nevertheless, these non­specific drugs are frequently deficient or not tolerated and do not target the underlying molecular mechanisms [140]. On the other hand, invasive surgical therapy can successfully benefit patients with drug resistance [140]. However, it can involve risks due to the nature of procedures and requires expertise that is not commonly available [141].

Finding a new therapeutic approach that targets the underlying mechanisms of DHF, such as gene therapy [142] or drugs that specifically target/inhibit specific pathways leading to DHF, would be invaluable in treating DHF. Mavacamten is an example of a recently available drug that suppresses cardiac myosin ATPase by diminishing actin-myosin cross­bridge formation. As a result, it addresses the underlying pathophysiology of hypertrophic cardiomyopathy [119], decreasing contractility and enhancing myocardial energetics [143]. In addition, because Mavacamten is a cardiac myosin inhibitor, it improves ventricular compliance and favorably impacts diastolic function [140, 144].

Other recently suggested DHF treatments include sodium-glucose co-transporter 2 inhibitors (SGLT2is) and mineralocorticoid receptor antagonists. These medications have shown promise in managing DHF, although the precise mechanisms of action are not fully understood. SGLT2is, such as empagliflozin, can benefit DHF management by promoting euvolemic diuresis, improving hemodynamics, and stabilizing heart function. A randomized, double-blind clinical trial was orchestrated to investigate the hypothesis surrounding SGLT2 inhibitors and their potential to mitigate the risk of hospitalization involving a cohort of 5988 patients divided between the treatment and placebo groups. The empirical data revealed a noteworthy discrepancy. The total occurrences of heart failure-related hospitalizations were notably fewer in the empagliflozin-administered group (13.8%) compared to the placebo group (17.1%). These findings significantly enhanced the condition of patients treated with empagliflozin compared to those in the control (placebo) group [145, 146]. Also, another clinical trial showed a decrease in the combined risk of cardiovascular death or hospitalization for DHF patients [147].

Mineralocorticoid receptor antagonists, which counteract aldosterone’s effects on water and electrolyte balance, have remarkably reduced morbidity and mortality among patients with congestive heart failure and left ventricular dysfunction [148]. Recent research highlighted spironolactone’s positive impact on LV diastolic function and myocardial fibrosis regression [149]. In addition, in DHF patients, using mineralocorticoid receptor antagonists was associated with reduced heart failure hospitalizations [150, 151]. In addition, *S*-glutathionylation of cMyBP-C is an essential alteration in cross-bridge kinetics and, accordingly, the development of DD. Therefore, sphingosine-1-receptor modulator FTY720 can partially reverse established DD and reduce left atrial enlargement connected with decreased oxidative alteration of cMyBP-C; it may help DHF [152, 153]. Thus, understanding and targeting the underlying mechanism of DHF can help develop effective pharmacological therapy for DHF, which is a significant unmet need.

## Conclusions

The management of DHF demands potential novel drugs beyond the existing treatments. Based on pre-clinical studies of DHF in transgenic models of myofilament disease, new approaches to intervention should focus on myofilament signaling, mitochondrial dysfunction, and myofilament structure/function. Deeper investigations will be essential to unveil the core mechanisms driving DHF, coupled with the development of animal models mirroring DHF symptoms. Such studies are key to identifying fresh therapeutic avenues targeting novel DHF treatment targets.

## Abbreviations

β-MHC: β-Myosin heavy chain
BP: Blood pressure
Ca^2+^: Intracellular calcium
CaM: Calmodulin
CPI-17: C-Kinase-potentiated protein phosphatase 1 inhibitor of 17 kDa
DHF: Diastolic heart failure
DD: Diastolic dysfunction
DHPR: Dihydropyridine receptor
ECG: Electrocardiogram
ELC: Essential light chain
FHC: Familial hypertrophic cardiomyopathy
HF: Heart failure
K^+^: Potassium
MLCK: Myosin light-chain kinase
MLCP: Myosin light-chain phosphatase
Na/K: Na^+^/K^+^ ATPase pump
NCX: Sodium–calcium exchanger
PLN: Phospholamban
RLC: Regulatory light chain
RyR2: Ryanodine receptor 2
SERCA2: Sarco/endoplasmic reticulum Ca^2+^-ATPase
SR: Sarcoplasmic reticulum
TD: Tissue Doppler
TnI: Troponin I
TnT: Troponin T
TnC: Troponin C
Tm: Tropomyosin
T-tubule: Transverse-tubule
SERCA-2: Sarco/endoplasmic reticulum Ca^2+^-ATPase
